# Pityriasis Rosea-Like Eruption following anti-fatigue traditional herbs: *Aconitum carmichaelii Debx* and *Panax Ginseng* suspected

**DOI:** 10.1186/s12906-024-04556-5

**Published:** 2024-06-29

**Authors:** Xueyan Zeng, Xin Zhou, Aiping Zhang, Yanqin Zhu, Bin Lu, Feiqin Zhu, Mengqi Wu, Riyang Lin

**Affiliations:** 1 Department of Traditional Chinese Medicine, Hangzhou TCM Hospital of Zhejiang Chinese Medical University, Hangzhou City, Zhejiang Province 310007 China; 2 Zhuji Fourth People’s Hospital, Zhuji city, Zhejiang Province China; 3 Key Laboratory of Kidney Disease Prevention and Control Technology, Hangzhou City, Zhejiang Province 310007 China

**Keywords:** Adverse effect, Traditional Chinese medicine, Pityriasis Rosea-like eruption, Anti-fatigue, Case report

## Abstract

Traditional herbs have a history of clinical use in anti-fatigue. However, several adverse effects of herbs have been identified. Pityriasis rosea-like eruption (PR-LE) is a rare cutaneous complication of herbs. To the best of our knowledge, there have been few reports of PR-LE following herbs. Here, we described a case of PR-LE that developed 6 days after taking anti-fatigue herbs. After the 17 days of stopping *Aconitum carmichaelii Debx* and *Panax Ginseng*, it notably faded. So, when anti-fatigue herbs being authorized for fatigue use, monitoring for potential adverse effects is necessary.

**Supplementary Information:**

The online version contains supplementary material available at 10.1186/s12906-024-04556-5.

## Introduction

Pityriasis rosea-like eruption (PR-LE) is a cutaneous complication associated with a number of medications and vaccinations. PR-LE is characterized by mildly inflamed, oval, papulosquamous lesions in the proximal regions of the trunk and extremities. Fatigue is a common psychophysiology disease, which is complex and involves multiple factors [1–3]. Traditional herbs have a long history of clinical use in anti-fatigue and are believed to have a well-defined efficacy and excellent safety [4]. However, it cannot be denied that traditional herbs can sometimes cause some adverse reactions, and the adverse effects of anti-fatigue herbs have not been fully characterized yet. Here, we report a case of PR-LE in a patient following anti-fatigue herbs.

## Case description

**Fig. 1:**
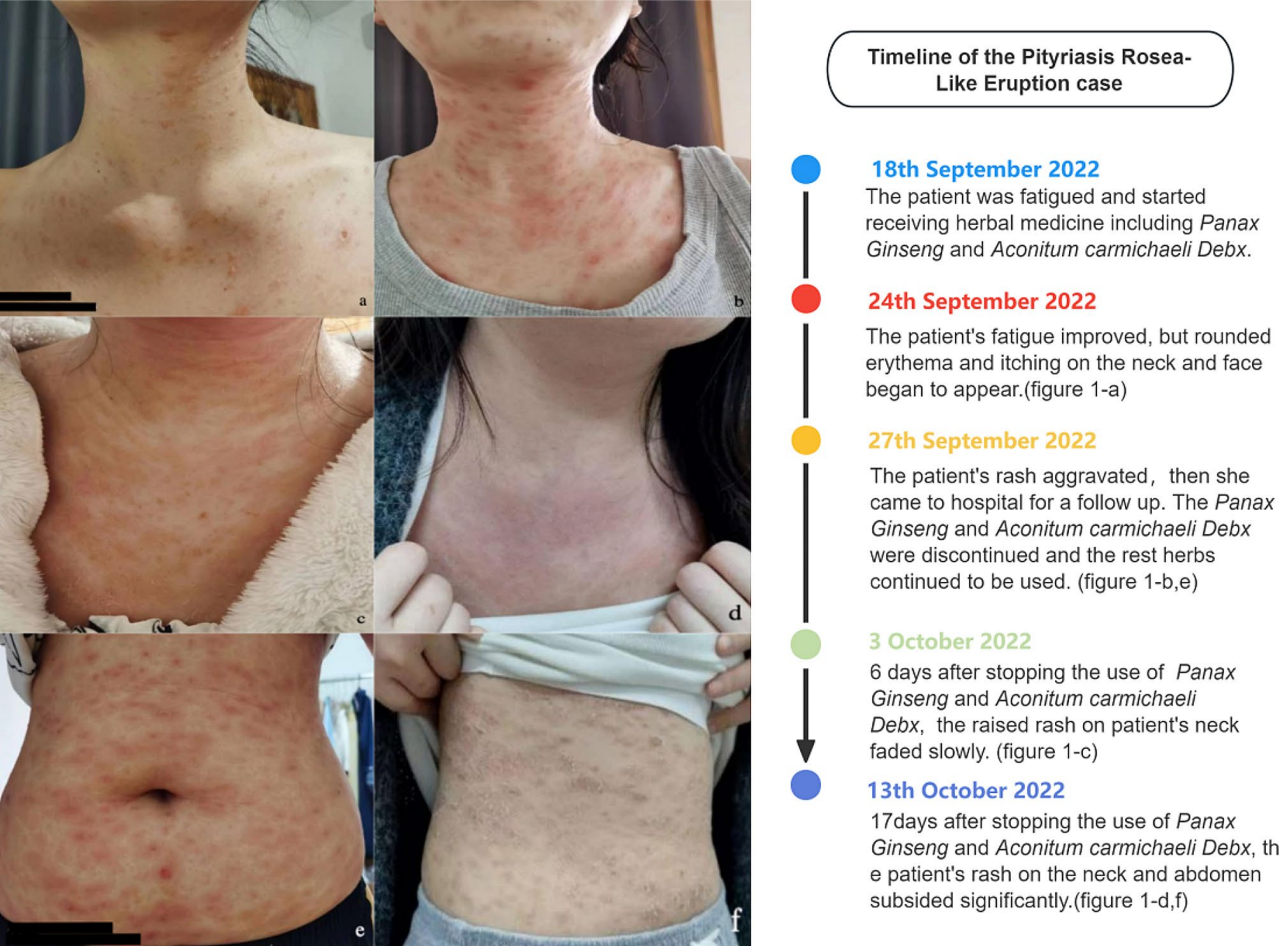
The photos and timeline of the Pityriasis Rosea-Like Eruption case

A 26-year-old female patient with fatigue attended the Hangzhou Hospital of Traditional Chinese Medicine on 18 September 2022. The patient was treated with Traditional herbs: *Aconitum carmichaelii Debx*(root), *Panax Ginseng*(root), *Cassiabarktree Twig*, *Chinese Goldthread Rhizome*(root), *White Peony Root*, *Liquorice Root*, *Fresh Ginger*(root). These herbs were boiled with water into 400 ml decoctions by the patient herself and then taken orally twice a day. The fatigue was significantly reduced within 1 week. However, this patient presented with an extremely itchy exanthem and oval erythematous lesion appeared on the neck and face since 24 September 2022 (Fig. 1-a). Rashes on the neck and face developed further and involved the abdomen (Fig. 1-b, e), then the patient came to a follow-up visit on 27th September 2022. There was no accompanying systemic symptom. The patient denied any history of allergies, recent infections, drug exposure or changes in lifestyle or diet, as well as similar skin rashes in personal or family history. So, Pityriasis rosea-like eruption was suspected. But the patient refused to take dermatological examination or any antihistamine or corticosteroid neither orally nor topically. *Aconitum carmichaelii Debx* and *Panax Ginseng* were discontinued, but the other herbs were retained to continue the treatment. Raised rash on the neck slowly flattens out after 6 days (Fig. 1-c). Eventually, 17days after stopping the use of *Panax Ginseng* and *Aconitum carmichaelii Debx*, the patient’s rash subsided significantly (Fig. 1-d, f). Moreover, the patient reported that the skin was in better condition than before the rash appeared.

## Discussion

Pityriasis rosea is an acute, self-limiting skin disease that resolves spontaneously. The exact cause of pityriasis rosea is elusive, and a number of infectious and non-infectious causes have now been proposed, including viral pathogens, vaccines and drugs. According to relevant reports, Drug-induced PR-LE accounts for 2% of all cutaneous adverse drug reactions [5]. Common causes of drug-induced PR-LE include angiotensin-converting enzyme inhibitors, non-steroidal anti-inflammatory drugs, and gold. It often presents with diffuse and confluent severely pruritic dusky-red erythematous lesions in the absence of herald patch [6]. Involvement of the oral mucosa has also been reported in as many as 50% of all cases [6].

Fatigue is a long protracted chronic disease that cannot be easily cured within a short time [7]. Given aging of the global population and the accelerating pace of life, fatigue incidences are increasing annually [8]. Fatigue is an early signal of related diseases but can also be the sequelae of multiple complex diseases. The World Health Organization (WHO) has declared that fatigue is a major risk factor for human life and health [9]. The COVID-19 pandemic aggravated the risk of fatigue [10, 11]. A large study involving more than 40,000 COVID-19 patients confirmed that a majority of cases (> 80%) presented at least one symptom 4 weeks after being diagnosed [12]. Among the symptoms, fatigue was the most common, occurring in up to 58% of patients. Currently, fatigue patients often require long-term treatment, the adverse effects associated with long-term administration of drugs cannot be ignored [13].

*Aconitum Carmichaelii Debx* significantly improve cardiac function, relieve pathological fatigue, and excite the nervous system, which is commonly used to treat heart failure and depression [14, 15]. *Aconitum Carmichaelii Debx* has a satisfactory effect on depression-like behavior of cancer related fatigue, which was related to the inhibition of neuroinflammation [16]. In many countries, especially East Asian countries such as China, Korea, and Japan, people believe that *Panax Ginseng* is the king of herbs because of its long history and various pharmacological activities. Nowadays, *Panax Ginseng* is received increasing attention as a kind of anti-fatigue product with obvious efficacy and fewer side effects [17–19]. *Panax Ginseng-Aconitum Carmichaelii Debx* herbal pair, is a classic traditional Chinese medicine (TCM) combination which has been widely used to treat fatigue and weakness [20].

In this case, the patient developed a rash six days after using a herbal tonic that included *Panax ginseng* and *Aconitum carmichaelii Debx*. However, at the same time, the patient felt that her fatigue was significantly better than before. Therefore, she considered the herbs are useful and wished to continue taking the herbal treatment. In traditional Chinese medicine theory, *Aconitum carmichaelii Debx* (which named *Fuzi* in Chinese) and *Panax Ginseng* (which named *Renshen* in Chinese) can warm yang and benefit qi, but too much yang and qi can turn into a fire-heat evil, which can cause rashes, fever and other illnesses. That’s why we considered stopping these two herbs after the rash appeared. Surprisingly, on the 17th day after stopping the two herbs, the rash subsided significantly, confirming our suspicions.

To the best of our knowledge, few herb-induced PR-LE has been reported. Here, we report the first case of PR-LE when taking *Aconitum carmichaelii Debx* and *Panax Ginseng*, but recovered after discontinued. PR-LE have similar presentation to pityriasis rosea, but our patient never experienced the prodromal symptoms, which are present approximately in 69% of typical PR [21]. The lesions of our patient were more itchy, diffuse and confluent than typical PR, resolved after *Aconitum carmichaelii Debx* and *Panax Ginseng* were discontinued [22]. The diagnosis is based on clinical and physical examination findings. The patient had no history of food allergies and denied taking other medications. We prescribed 3 g *Aconitum carmichaelii Debx* and 9 g *Panax Ginseng per* day to this patient, both at safe doses [23, 24]. Although the exact pathophysiologic mechanisms of PR-LE after herb-taking remain unclear, an autoimmune response is a possible etiological factor, and many studies have shown changes in the autoimmune response in patients with PR-LE [25, 26]. *Aconitum carmichaelii Debx* has been shown to boost autoimmunity, and Aconitum carmichaelii polysaccharides can increase white blood cells and lymphocytes, reverse the decreased mRNA expression of NF-кB, IL-6, and iNOS, differentiation of CD4 + FOXP3 + regulatory T cells as well as protein expression of occludin and zonula occludens [27]. Thus, we speculate that stimulation of the immune system induced by *Aconitum carmichaelii Debx* have contributed to the etiology of PR-LE. However, according to available studies, ginseng significantly decreases the pro-inflammatory cytokine interleukin (IL)-1 and significantly increases the anti-inflammatory cytokine IL-10, suggesting that it has anti-inflammatory effects [28, 29]. So, the possible pathological mechanisms of ginseng-induced PR-LE need to be further researched and investigated.

There are several limitations in this case report. First, our patient was diagnosed based on clinical and physical examination findings, histopathological examination lacked due to refusal of our patient. Second, for medical ethical reasons, we could not reintroduce the patient to these two herbal remedies in order to verify whether similar adverse effects would recur. So, it is not possible to identify either *Aconitum carmichaelii Debx* or *Panax Ginseng* was responsible for PR-LE in this case. Although *Aconitum carmichaelii Debx* seems to have more adverse effects, further studies are necessary. Third, it seems that herb-induced PR-LE recovered within 17 days after discontinuing *Aconitum carmichaelii Debx* and *Panax Ginseng* as our patient refused to use antihistamine or corticosteroid. However, it should be noted that our patient continued to take other herbs (*Cassiabarktree twig*, *Chinese Goldthread Rhizome*, *White paeony root*, *Liquorice root* and *Fresh Ginger*), which may have facilitated her recovery.

## Conclusion

In summary, we have described a rare report of PR-LE, the etiology may be related to either herb-induced stimulation of the immune system, or some rare herb component. Analysis of the case using the Naranjo adverse drug reaction probability scale indicated that *Aconitum carmichaelii Debx* and *Panax Ginseng* were likely to be the causes of the pityriasis rosea-like eruption (Supplementary material 1). We believe such a case is unique and it has not been reported previously. Fortunately, PR-LE is responsive to discontinuing *Aconitum carmichaelii Debx* and *Panax Ginseng* without any systemic adverse effects. Our report highlights that the presence of PR-LE is not a contraindication for subsequent anti-fatigue herb treatment. However, when anti-fatigue herb being authorized for fatigue use, monitoring for potential adverse effects (e.g., cutaneous reactions) is necessary.

## Electronic supplementary material

Below is the link to the electronic supplementary material.

Supplementary Material 1

Supplementary Material 2

## Data Availability

All data generated or analyzed during this study are included in this published article.

## Abbreviations

PR-LE: Pityriasis rosea like eruption
TCM: Traditional Chinese Medicine
