# Correction: Pityriasis Rosea-Like Eruption following anti-fatigue traditional herbs: *Aconitum carmichaelii Debx* and *Panax Ginseng* suspected

**DOI:** 10.1186/s12906-024-04665-1

**Published:** 2024-10-18

**Authors:** Xueyan Zeng, Xin Zhou, Aiping Zhang, Yanqin Zhu, Bin Lu, Feiqin Zhu, Mengqi Wu, Riyang Lin

**Affiliations:** 1 Department of Traditional Chinese Medicine, Hangzhou TCM Hospital of Zhejiang Chinese Medical University, Hangzhou City, Zhejiang Province 310007 China; 2 Zhuji Fourth People’s Hospital, Zhuji city, Zhejiang Province China; 3 Key Laboratory of Kidney Disease Prevention and Control Technology, Hangzhou City, Zhejiang Province 310007 China

**Correction:** ***BMC Complement Med Ther*** **24, 248 (2024)**

10.1186/s12906-024-04556-5

Following publication of the original article [1], the authors reported an error in affiliations. The primary affiliations in the paper must include both the name of the university and the hospital.

The author group has been updated above and the original article [1] has been corrected.
