# Supplementary material for: Pityriasis Rosea-Like Eruption following anti-fatigue traditional herbs: Aconitum carmichaelii Debx and Panax Ginseng suspected
Source: BMC Complement Med Ther. 2024 Jun 29;24:248. doi: 10.1186/s12906-024-04556-5 (PMC11218121; doi:10.1186/s12906-024-04556-5)
Supplement: Supplementary file 1 — Supplementary Material 1 [file 12906_2024_4556_MOESM1_ESM.pdf]

Naranjo Adverse Drug Reaction Probability Scale

Question	Yes	No	Do Not Know	Score
1. Are there previous *conclusive* reports on this reaction?	+1	0	0	0
2. Did the adverse event appear after the suspected drug was administered?	+2	-1	0	2
3. Did the adverse reaction improve when the drug was discontinued or a *specific* antagonist was administered?	+1	0	0	1
4. Did the adverse event reappear when the drug was re-administered?	+2	-1	0	0
5. Are there alternative causes (other than the drug) that could on their own have caused the reaction?	-1	+2	0	2
6. Did the reaction reappear when a placebo was given?	-1	+1	0	0
7. Was the drug detected in blood (or other fluids) in concentrations known to be toxic?	+1	0	0	0
8. Was the reaction more severe when the dose was increased or less severe when the dose was decreased?	+1	0	0	1
9. Did the patient have a similar reaction to the same or similar drugs in *any* previous exposure?	+1	0	0	0
10. Was the adverse event confirmed by any objective evidence?	+1	0	0	0
**TOTAL SCORE:**				6

Modified from: Naranjo CA et al. A method for estimating the probability of adverse drug reactions. Clin Pharmacol Ther 1981; 30: 239-245.
